# Ketamine-assisted psychotherapy, psychedelic methodologies, and the impregnable value of the subjective—a new and evolving approach

**DOI:** 10.3389/fpsyt.2024.1209419

**Published:** 2024-02-01

**Authors:** Phil Wolfson, Gita Vaid

**Affiliations:** The Ketamine Research Foundation, San Anselmo, CA, United States

**Keywords:** ketamine, psychotherapy, psychedelics, alternative medicines, subjectivity, consciousness, psychiatry

## Abstract

Psychiatry is in a growth phase in which several psychedelic medicines have entered its arena with great promise. Of these, presently, ketamine is the only medicine that may be legally prescribed. We hypothesize that at subanesthetic doses, ketamine produces a unique spectrum of altered states, ranging from psychoactive to deep ego-dissolving experiences, that are intrinsic to ketamine’s therapeutic effects. When these experiences are embedded in a therapeutic relationship—a setting—that fosters an amplification of the recipient’s subjective consciousness, personal growth, inner healing, greater clarity, and better relationships may well ensue. While much of the literature on ketamine labels its dissociative effects as ‘side effects’, alteration of consciousness is a component and unavoidable ‘effect’ of its therapeutic impact. From its inception in the clinical trials of the 1960s, ketamine was recognized for producing dissociative, psychedelic effects on consciousness in subjects as they emerged from ketamine-induced anesthesia. Unanticipated and unintegrated, these experiences of ‘emergence phenomena’ were felt to be disturbing. Accordingly, such experiences have been typically labeled as dissociative side effects. However, in a conducive set and settings, these experiences have been demonstrated to be of positive use in psychiatry and psychotherapy, providing a time-out from usual states of mind to facilitate a reshaping of self-experience along with symptomatic relief. In this way, ketamine-assisted psychotherapy (KAP) offers a new potential in psychiatry and psychotherapy that is powerfully valanced toward recognizing experience, individuality, and imagination. Essential to a successful therapeutic experience and outcome with KAP is close attention to the subjective experience, its expression by the recipient and integration of the ketamine experience as a healing opportunity.

## Introduction—KAP, values, and psychotherapy

In essence, given the complexity of human beings and their consciousness and sufferings, it is essential to attend to the primary data—our subjective experiences (1, 2).

The countercultural movements of the 1960s and 1970s effectively brought attention to the benefits of pleasure and well-being in a reversal of the suppression that preceded it. Exploring the unity of mind and body and informally treating trauma and emotional distress with medicines that produced altered and expansive states of consciousness, the use of these medicines contributed to enlarging the personal and social sense of freedom, equality, and openness. Mass use grew exponentially and has been worldwide, despite criminalization. The scheduling of psychedelic medicines blocked this freedom to explore. It occurred in a draconian manner, with prohibitions against even investigatory human research with psychedelic medicines. This resulted in a massive incarceration for even possession of small amounts of these medicines and an expansion of police powers and the prison system. The so-called War on Drugs could be seen as a war against cultural impulses that threatened entrenched hierarchies of political and social control. It stopped the flowering of a new and liberating vector in psychiatry that focused on the positive aspects of being alive. It also served the emergence of big pharma (3–9). It suppressed and repressed our primary data, the subjective nature of human beings (10–12).

In the last decade, an opening to the clinical potentialities of alternative medicines with psychedelic—mind manifesting—properties is in process (13, 14). A vast public use of psychedelic medicines has and is occurring (15), and the realization of their safety (16) and benefits has resulted in a mass movement to decriminalize, this best exemplified by marijuana’s ever-increasing access. Recently, there have been varying levels of decriminalization of psychedelics in states and localities. Clinical research with two Schedule I substances—MDMA and psilocybin (extracted from mushrooms)—appears headed for approval for clinical prescription on successful completion of Phase 3 FDA-approved studies. On approval, the FDA’s limitations regarding methods of use —Risk Evaluation and Mitigation Strategies (REMS)—will be forthcoming. The resultant cost to patients will be determined, and financial factors will control access to their use (17).

For a variety of reasons, but principally because of its safety, its indication and use as an anesthetic, and its widely distributed administration, ketamine escaped suppression as a powerful psychedelic medicine. Its off-label use has been largely unregulated save for the general Drug Enforcement Administration (DEA) requirements. Ketamine is an FDA Schedule 3 substance dating from 1970 with an indication for anesthesia and historically in use as an analgesic as well. With this classification, anesthesiologists have had control of ketamine and have extended their reach to psychiatric applications—particularly depression—with their attendant clinics. As anesthesia itself is a broad modality, off-label use is not limited to a particular diagnostic entity. No specific diagnosis is required under the necessary flexibility of its anesthesia indication. This has fostered exploration of ketamine’s application to a broad range of diagnostic entities, especially by psychiatrists.

The ketamine molecule arose in the search for an alternative to phencyclidine (PCP), which has powerful anesthetic properties, is hallucinogenic, and became associated with individuals manifesting violent behavior (18). Immediately, within the workup for FDA, in the 1960s (19), disturbing hallucinatory experiences were noted as metabolism of ketamine occurred, and the anesthetic state diminished. Recipients were unprepared and confused. These were felt to be undesirable properties of the new medicine and were dubbed the ‘emergence phenomenon’ in reference to what occurs with the lessening of the anesthetic effect. Consequently, there was no patient education or preparation for this possibility, despite ketamine’s wide use from 1970 on. However, this information reached individuals interested in psychedelic medicines, and the use of ketamine for its particular powerful mind-altering effects began [See Jansen (20) and Wolfson and Hartelius (1) for this history]. While no studies have compared the dissociative effects between PCP and ketamine, both molecules produce dose-related profound alterations of consciousness, ketamine without the violence and of shorter duration—2.2 h half-life for ketamine versus 21 h for PCP (21).

In the late 1990s, ketamine started to be explored in academia—inpatient psychotic patients at first (22), where it had no impact on psychotic symptoms, but attention was brought to its beneficial reduction of depressive symptoms. These noteworthy findings attracted research and investigation for clinical use. With the antidepressant effect, also came what were called its dissociative effects (the emergence phenomena). From the beginnings of its psychiatric application, ketamine’s psychedelic properties have been found to be coterminous with its antidepressant effects. This linkage presented a challenge to ketamine’s use psychiatrically given that the War on Drugs had instigated such prejudice against psychedelics that their development was completely suppressed.

Despite the perceived challenge of minimizing or suppressing the dissociative state that accompanies ketamine’s antidepressant effects, ketamine showed promise in the treatment of depression in early clinical studies beginning in 1999 (22, 23). This new entrant was welcomed in part due to conventional medicine’s lack of success with a third or more of patients with so-named treatment-resistant depression (TRD), defined as failing to respond to adequate trials of two or more antidepressants. A positive new entrant into the treatment of the massive number of people suffering from depression had vast possibilities for benefit and endlessly generated economic potential. Clearly, the causes of significant depressions were and are not abating, but rather are actually increasing due to social and environmental stress (24–26). Conventional psychopharmacological psychiatry had fallen on hard times (14). The failure of antidepressant treatment has been obvious to psychiatry for decades, and there has been no new significant introduction by Pharma for a similar period (27, 28).

A new and different healing modality would clearly be of value. Ketamine’s main issues for conventional psychiatry and the treatment of TRD would be the attendant altered states that accompany its antidepressant effects and how to minimize or suppress awareness of that linkage.

Three basic strategies resulted to cope with the reality that ketamine has dissociative/psychedelic properties—.

Doses as low as possible with preservation of some degree of antidepressant efficacy [arbitrarily chosen as 0.5 mg/kg delivered over 40 min intravenously (IV)]. Note that in practice and driven by the need for its efficacy, IV doses have steadily climbed to as high as 2.0 g/kg in some instances (29–31).

IV administration: The intravenous route of administration keeps ketamine in the hands of anesthesiologists and psychiatrists and confined to clinical settings that require medical administration and supervision, where no psychotherapy is offered. This context may mislead patients by implying through contextual cues that the therapy is strictly medical and that ketamine has no psychological implications or emotional impact at this low dose. Adding to this has been the lack of psychiatric and psychotherapy training of ketamine-administering anesthesiologists, this bearing on their selection of patients and predominantly choosing those with TRD (as if this itself is an easy and narrow diagnostic entity).

Denial—Deliberate omission of reference to, or understatement of, psychedelic effects, or dismissing these as undesirable or irrelevant side effects.

Unsurprisingly, patients who experience and report psychedelic effects in such contexts have no preparation or support to process or understand them, which they may then find concerning, likely adding to the sense that these effects are undesirable. Yet several papers by major research groups have indicated that psychedelic effects are the variable that correlates most closely with outcomes (32–36). Moreover, numerous studies show that a sustained antidepressant effect is only achieved with multiple ketamine administrations, six infusions over two to three weeks becoming the norm, a protocol that necessarily entails higher cumulative dosage and increased duration of psychedelic effects (37, 38). It is clear that dosage and positive outcomes increase together, regardless of whether dosage is considered per treatment or cumulatively.

In a conventional research context, this correlation between psychedelic effects and therapeutic outcomes would point to the former and their concomitant neural activity as the likely therapeutic factor. Despite this, the focus of current research is less on uncovering how psychedelic effects are implicated in benefit than on how to obtain those benefits without the psychedelic effects. This approach assumes, apparently based on bias rather than evidence, that these effects are undesirable and that they are separable from the beneficial impact.

Central to this discussion is Ballard and Zarate’s 2020 meta-analysis of dissociative effects in the treatment of depression with ketamine (34). To begin with, every study included in this meta-analysis includes reference to dissociative ‘side effects,’ a fact fully consistent with the conclusion that altered states of consciousness are an essential part of ketamine’s therapeutic effect. If there is no impact on depression absent some degree of altered state, then ketamine-induced psychedelic experiences are part of ketamine’s effect and not a side effect.

The association between psychedelic and therapeutic effects is all the more likely given that in the above-cited meta-analysis (34) and other reviews (36–39), all efforts to synthesize an N-methyl-D-aspartate receptor (NMDAR) antagonist, the most likely mechanism of action of ketamine (40–42), that produces an antidepressant response without dissociative effects have failed. There is also some evidence that ketamine acts on one specific type of NMDAR that is associated with both psychoactive and antidepressant effects. While there are myriad types of NMDARs, only a few have been characterized. In one of these, the GluN1a-GluN2B Cyro-EM structure, ketamine binds to the same site where PCP molecules attach, the so-called PCP site (43). If ketamine binds selectively to this site, then the psychoactive effects may be biologically inseparable from its impact on depression.

Despite ketamine’s promise, it is not clear that the most common treatment protocols show impressive benefits. These are characterized by limited dosage, IV administration, short treatment duration, and the absence of psychotherapeutic engagement. A recent meta-analysis by McInnes et al. (44) demonstrated a success rate of 28.9%, which belies claims of great utility.

From a review of 9,016 patients, 537 had sufficient data for inclusion. For these patients treatment consisted of 4–8 infusions over 7–28 days; results showed that 53% responded and 28.9% remitted. Responders had about an 80% probability of sustaining their response at 4 weeks and 60% at 8 weeks. Fifty-four percent of patients completing initial treatment elected to enter a maintenance phase consisting of 3–4 sessions. The full length of maintenance was not reported but appeared to be greater than 80 days from the commencement of treatment. The modest remission rate can be seen as pointing to the need for adjustments to the conventional protocol in ways that have shown promise: higher doses administered without the expense of medicalized contexts, maintained over longer periods, and paired with psychotherapeutic intervention. We have concluded that, in essence, this study provides a reference for the differentiation of KAP from unsupported IV administration; this is to be referenced as KAP’s own analyses proceed.

The previously cited meta-analysis (44) also illustrates an underlying issue: the stance of most ketamine studies is pharmacological, not psychological, emotional, and relational. Curiosity about the potential therapeutic impact of ketamine’s subjective effects is entirely lacking. Evidence for this question is constrained by the fact that the studies reviewed utilize dosages designed to limit these subjective effects—only one has a range from 0.1 mg/kg up to 1.0 mg/kg. When study design intentionally restricts the dissociative effects of ketamine, evidence for possible relationships between ketamine’s subjective effects and its efficacy as an antidepressant is obscured.

An effective study of this relationship also requires the use of an appropriate measure. The Clinician-Administered Dissociative States Scale (CADSS) was developed as a diagnostic measure for dissociative disorders (45) and tends to underreport the qualitative differences in states associated with ketamine. This same criticism has been made, for example, with the use of the CADSS with the esketamine protocol (46). Pahnke’s Mystical Experiences Questionnaire (MEQ) (47) and the Ego-Dissolution Inventory (EDI) (48) are more aligned first-person report measures for assessing beneficially altered states of mind.

The fact that ketamine is a non-specific medicine shown to have positive effects on a broad spectrum of disorders such as bipolar depression, post-traumatic stress disorder, obsessive-compulsive disorder, suicidality, and relational distress is further evidence that its effects are not merely pharmacological (49). This is typical of compounds with psychedelic effects: benefits are not confined to specific diagnoses, even though they may have direct therapeutic impact on specific symptoms associated with conditions such as trauma and depression. Due to these broad-spectrum impacts on subjective experience, Ballard and Zarate’s proposal that ketamine, psilocybin, and MDMA constitute a class of drugs that should be considered rapid-acting antidepressants (RAADs) (27) is a misnomer that considers only one dimension of their healing effects on conscious experience.

The view that ketamine is a specific medication for TRD is more a reflection of the mindset of current psychiatry and the mechanisms of FDA approval of prescriptive use for Schedule I substances than it is an accurate characterization of ketamine’s therapeutic potentials. Millions of people continue to suffer from depression in a broad spectrum of formats, and ketamine offers an opportunity to provide relief for many of these variations. All psychedelics, including ketamine, show broad effects on mind and body and require a view that acknowledges efficacy for specific diagnoses without limiting these pluripotent substances to any one application (28).

Hailing ketamine treatment as a new milestone in the treatment of depression is likely appropriate, but its potentials are not reflected in the low rates of remission seen in many studies [e.g., (44)]. Further demonstration of ketamine’s pharmacological effects is needed, a project that will be aided by differentiating its biological and psychological impacts. There are a number of experiential aspects that may contribute to ketamine’s efficacy, such as the break from one’s usual mindset—often experienced as a time-out for new beginnings and renewed visions, aided by the way in which its dissociative effects may expand the imagination. The experience of letting go of control also appears to have impact on patients, allowing for a freedom of inner exploration in ways that may also affect processes of being in the world. Ketamine may facilitate the type of inner healing associated with disruptions in consciousness with treatments such as prolonged sleep inductions and electroconvulsive therapy—research that has been pursued for a century. There is a great need for clinical research directed toward careful examination of these potentials.

With a low percentage of positive remissions from IV administration as above (44), ketamine’s specificity of effect is further confused. Untangling the psychological impact of ketamine from its pharmacologic activity is a work in process. Indeed, there are many possibilities for ketamine’s effects. In our experience, a major impact is the break from our usual mindset, a time-out for reformation and starting anew, and the expansion of imagination caused by dissociative effects. The positive aspects of letting go of control that occurs with ketamine experiences no matter the route of administration appears to be singularly impactful—engendering a freedom for exploration of mind and being in the world. A ketamine experience may well relate to the process of Inner Healing. It may be a matter of the degree of disruption of consciousness that psychiatry has been exploring for a century with ECT and prolonged sleep inductions. Further delineation of ketamine’s direct pharmacological effect is a necessity. Clearly, both subjective effects and pharmacology are involved. Our interest is in providing clinical research that is directed toward a critical elaboration of these potentialities.

## Ketamine, psychedelics, and the subjective as the essence

Ketamine is an ideal medicine for clinical use as an assisted psychotherapy. Its particular characteristics including duration of action enables effective applications in office settings. This was first delineated by the Australian psychiatrist Stephen Hyde in a presentation at the initial KRIYA Conference on ketamine in 2015 and then in his 2016 *Ketamine and Depression* book (34). Hyde challenged the mode of medicalized IV administration of by demonstrating the safety of ketamine in an office setting using oral buccal administration accompanied by psychotherapy. This fostered ketamine’s application to comfortable non-medical settings. This non-invasive administration enhanced his outcomes as did the provision of a full psychiatric approach to patient care and to patients’ subjective experiences. *The Ketamine Papers* furthered the development of ketamine’s administration as an assisted psychotherapy and especially at the subanesthetic doses used in psychiatric/psychotherapy settings, (1) Focus and emphasis on the value of the dissociative state have made its use in office and in supervised at-home sessions valuable additions to the expanding possibilities for alternative medicines as a shift in the psychiatric paradigm, (2) Ketamine provides an immediate effect, great potency for eliminating active suicidality (50, 51), intermittent use, minimal side effects, and short duration of action. Our conclusion has been that it is an ideal psychedelic medicine for clinical use.

Ketamine is one of the most powerful psychedelic medicines in existence (1, 20). To treat ketamine as a drug and administering it in medicalized settings without providing a witness to the expression of the experience leaves patients in the hole of ‘what happened to me?’ Patients become disoriented and confused and left with a self-contained explication—essentially sorting it out by oneself. And from the clinician’s point of view, there is reliance on a drug effect as if the drug being administered is the same as conventional antidepressants. Many patients experiencing this as a ‘procedure’ express feeling left high and dry (52). Especially for altered state-naïve patients, there is an emptiness of expression that is troublesome, leaving them in an inexplicable, uncommunicated, or validated realm of experience. Providing ketamine in this manner misses the opportunity for a therapeutic encounter that encompasses the infinite range of internal experience and that opens the door to personal change in a conscious as well as unconscious transformative process. Offering an alternative to the medical setting and situating KAP therapy in the office, a conducive setting for psychotherapy—a therapeutic nest ala Winnicott (53)—can and has been occurring since 2015.

The dialectic over the modes of administering ketamine—between the pharmacologic approach versus that of the interest in subjectivity—lies at the root of the divide. Certainly, it is easier to prescribe, stay distant, and walk away to the next patient than it is to come to know someone in all their complexity and to be engaged with them in their struggles of being and living. The move to Pharma took psychiatry out of its prior emerging interest in human beings and into prescription as its tour de force (54). In truth, the psychiatric toolbox, to the realization of those working in the field, is limited in both options and efficacy. And indeed, interest in becoming a psychiatrist has plummeted as interest in humans plummeted. This is rapidly changing with the movement toward prescriptive legalization of psychedelic medicines—a new enthusiasm is building (55). Currently, ketaminne is the only legally available psychedelic medicine--one with profound effects and an emerging set of methodologies that increase the potency of working therapeutically for the benefit of human beings as per KAP (56–58).

The subjective and its narrative reportage are fundamental to our examination of consciousness and mind (59). Extrapolation from studying brain function, as well as the tools and protocols used to do so, occurs through the subjective lenses of both the subject studied and the investigator. For example, fMRI studies require inputs arranged for effects on subjectivity to have those effects then observed in the machine. That observation is of subjective effects. Practitioner-based assessment tools deemed to be more accurate than personal reports include at least three levels of separate subjectivities: the creators of the tool, the administrator of the tool, and the recipient of the tool. Objectivity or canalized self-reportage (60)? This does not diminish the value of exploration but rather limits the accuracy of imputations. And this is reflected in the constant revision of neurophenomenological explanation in the literature.

Attention to our subjective nature is what makes us feel whole, loved, and connected. Absence and limitation of that attention are the sources of confusion and distrust about belongingness and connectedness. Powerful and novel experiences need to be shared for their integration into our manner of being ourselves and in the world. It is this being unto ourselves that allows for all manner of reception and evaluation of what comes into us and from us. Subjectivity means differentiation from everything and yet conscious and unconscious integration with everything. Our sensorimotor being is a format and a depiction of reality. We are interpreters from the moment life began with its separation to inside and outside.

The expression of our subjectivity is a voluntary thing, its fuller expression based largely on trust—and since that has been so damaged in so many of us, there is an art—the art of relationship creation–that is essential to repair, for realization, and happiness to value and thrive in this precious life (61). These are the desired outcomes of what therapies can deem as success. This occurs when our subjectivity is valued and engaged in relationship (62). This is the art of psychotherapy and of our work with ketamine. Having a profound journey or letting go of our protective layers at lower doses requires a relationship of trust and relaxation of our guard. Or the experience is attenuated or is even negative. ‘Setting’ aids our set—our emotional, spiritual orientation, our sense of friendliness, and our receptivity to having experiences that can include healing, awareness, expansion, and balancing. The literature on psychedelics is replete with this (1, 3, 4).

Ketamine experiences tend to have an ego-dissolving effect and to cast us into a sense of our smallness and our connectedness as part of larger wholes. They may engender a sense of the divine in us— and outside of us—and the preciousness and impermanence of this life. In this vein, there have been several studies of varying quality examining the relationship of psychedelic experience to progressive outlooks regarding prejudice and world views that suggest positive change, reduction of prejudice, and self-centeredness—narcissism (63–69). These manifestations of social consciousness emanating from the core of psychedelic psychotherapies are a part of the KAP inquiry into its effects, particularly regarding transformation and ego-dissolution—major components of KAP. They are not inevitable, but rather a tendency in the direction of balance and respect for others.

The quality and depth of the subjective experience is enhanced by a complex of components that are essential to the quality of psychedelic-assisted psychotherapies including KAP. They emanate from our clinical experience with thousands of KAP sessions in now thousands of KAP recipients and in collegial exchanges with hundreds of colleagues practicing therapeutically with KAP. These are presented in their essence and order in Table 1.

**Table 1:** Steps to enhance the subjective experience of ketamine therapy.

The primary preparation for the experience is the building of trust—in the therapist and in the ketamine process. This is abetted by knowledge of the patient’s life and struggles, and the creation of a zone of perceived safety and warmth. Ritual and the creation of a non-religious sacred setting enhance outcomes. Then—and it needs be said ‘only then’—comes the provision of the experience of altered consciousness—when the patient and the conditions are ripe for it. Next, is the observation by the recipient of that experience while it is occurring and as its narration is or becomes possible. This is often felt as a shift in self-awareness that occurs both consciously and as a release from usual struggles and obsessions—often referred to as ‘inner healing’ for its inherent subjective reconstruction of the self-experience—and its expression of this realization to oneself and to others. The consolidation of the experience comes with the meaning-making process by the recipient—in concert with the therapist, who helps to ‘anchor’ that which is noteworthy to stabilize change and awareness of change, this introjection serves as a rebalancing if and when recurrence occurs. This can be viewed as a return to the knowledge and memory of the vital self-experience of the ketamine journey. Follow-up and further processing, often referred to as Integration, are essential components of KAP. Collaboration between therapist and patient on the possibility of additional sessions. Inclusion of outside treating therapists and psychiatrists if they are involved in the patient’s treatment. Inclusion of family and relationship members as per the patient’s wishes.

## Ketamine science, psychedelic signatures, and the ketamine signature

The ketamine subjective experience has its own format based on its particular characteristics as an idiosyncratic response. All psychedelic medicines differentiate in certain and particular aspects, which we have come to refer to as their ‘Signatures’ (2). They also share many properties experientially. The concept of the ‘Signature’ arises as a frame for describing and delineating both ketamine’s particular characteristics and affording the heuristic potentiality for examining and specifying differences from other mind-altering medicines. It enables the creation of a subjective and behavioral array of ketamine’s properties that contrasts with the reductionistic nature of neuroscience.

Toward comprehending ketamine’s properties on multiple levels of exploration, neuroscience has evolved potential explanations for the array of ketamine’s effects on body and mind. Of course, this makes neuroscience both interesting and complicated. In evaluating what remain at best putative explanations of ketamine’s mechanisms of action, we contrast these with the generalization as to ketamine’s subjective experience for a sense of their accuracy and power of explanation.

In the classification of the characteristics of psychedelic medicines, there tends to be a reductionistic explanatory vector contained in such formulations as ‘classic psychedelics’, which apparently share 5HT_2A_R neurotransmission (70). Clearly, there is more to the story as they would not differentiate in their various effects if that were truly a unifying mechanism of action. Additionally, those dubbed ‘classical’ are in two separate chemical clusters—tryptamines and phenethylamines. As the serotonin hypothesis has long had its problems, neuroscience has increasingly focused on intracellular mechanisms of action for psychedelic effects. Add to this the glutamatergic system in its presence as 90 % of the neurotransmitters present in the brain compared with the 2 % for serotonin, and there is more difficulty in sustaining a single neurotransmitter hypothesis (71).

Higher levels of integration appear to offer more explanatory power as they are unveiled. Differentiation between alternative medicines occurs at the phenomenal level with the mobilization or suppression of differing activity and integrations; with the neural nets involved in receptor sensitization by the molecules ingested; and by the pluralism of the individual’s particular potentiation. This remains a science in its neonatal state. Assertions of definitive mechanisms are suspect, are in contradiction and revision, and are postulated at highly reductionist levels of explanation. The integration of felt experience and mechanistic explanation remains distant. To add to this conundrum, while rodents are accessible animals for studies, extrapolations to humans are being made from brains containing 30 million or so neurons to humans with 86 billion neurons. The difference speaks to a massive order of complexity (1, 72).

With respect to ketamine, there is an entirely different putative mechanism of action—the glutamatergic system. With dosage escalation, ketamine creates vivid hallucinatory experiences that are often full stories, or philosophical explorations visually, or cosmic, or even fractal for some—similar, or related experiences being particularly notable for psilocybin, 5 Meo-DMT, DMT and ayahuasca containing DMT in its admixture. This commonality of journey experiences crosses neurotransmitter explanations and invokes larger scale hypotheses for explaining effects in common (1, 3, 4, 73). In a steady stream, we tend to offer levels of explanation—molecular, structural, neuronal, mechanical, network, etc.—none of which is adequate to explain or enable the creation by artificial means of the subjective experiences encountered. Primary is experience itself, which is described as ineffable—meaning description in language internally or as expressed is an inadequate transcription.

It is alluring to embrace new neuroscience. Indeed, it is interesting and intriguing to explore and find new mechanisms, or more accurately hypotheses about new mechanisms. These levels of explanation loosely consist of the molecular neurochemistry, the representational—such as EEG and fMRI, the study of brain relationship networking, in other words a scaffold of partial and at times interrelated mechanistic explorations using the expanding tools of science embedded in investigator interests and perspective (See for example, Carhart-Harris’s Entropic Brain (73) and Pivotal Mental States (74)). The most prominent theory for ketamine’s mechanism of action involves the NMDA receptor to which ketamine is an antagonist, and this lies within the most widespread neurotransmitter presence in our brains—the glutamatergic system (31, 75)—as above.

While there has been a persistent attribution of mode of action to neuroplastic effects of ketamine, this is highly controversial, and the science is all over the map. In fact, as is becoming more obvious that axonal and dendritic responsivity and rearrangement is necessary for all forms of learning and the incorporation of experiencing. It is bivalent, that is responsive to both negative and positive experience. The calculation of the speed of neuroplastic movement is various depending on the research ranging from near instantaneous to many days for reformation (76–79). This deserves a full review on its own which is beyond the scope of this paper.

Further confusion about ketamine emanates from the ketamine molecule being stereoisomeric with its two enantiomers, the S(+) and the R (−) configurations, and being a hydrochloride salt. Understanding this leads to better clarity about claims made for different products and more informed prescribing. Historically, and in general, off-label use for psychiatric indications, as well as for anesthesia and analgesia, is an equimolar racemic mixture of the two enantiomers. Although racemic ketamine has the broadest worldwide use, S(+)-ketamine is available in some European countries and came to market as an FDA approved for TRD patented nasal preparation for psychiatric use in 2019—esketamine. Esketamine is considered to be 3–4 times more potent (19, 32, 80) than the R isomer (81), and at 50% of the content of the racemic RS material has about 75% of its potency. Comparisons of the relative antidepressant strengths of the RS IV use of ketamine versus the esketamine IN use are constrained by doses that are not comparable (82). In its general clinical use, while the esketamine preparation is rigidly controlled at a maximum and often ineffective dose of 84 mg, the IV, intramuscular (IM), sublingual (SL), and intranasal (RS -IN) routes are subject to dosage variability. In the Bahji et al. (81) comparison, it is the esketamine relationship to the IV 0.5 mg/kg dose that has been most reported and forms the basis for this inadequate comparison of effects (83).

As the hypothesis is explored, the nature of the response to ketamine is further defined by the manner of ketamine’s provision. There are three essential vectors that govern an individual’s response to ketamine:

- The route of administration, which variably governs the speed and amount of ketamine absorbed. These include topical creams, intravenous drip, intravenous bolus, intramuscular, sub-cutaneous, sublingual, nasal, oral, vaginal, and anal. Each has its own rate of onset of effects, quantity absorbed, and adherents, as well as a rationale for the route of choice. Focusing on mucosal absorption, and particularly the sublingual and nasal, the length of time held in the mouth or nose will determine the amount absorbed.
- The amount of ketamine administered.
- The sensitivity of the individual to the medicine: This varies widely and is not predictable by any available means. While mg/kg certainly influences the amount delivered by any route, it is not linearly related to the effect of ketamine on a recipient. This means that ketamine’s effects are determined empirically by clinical trial. The rule of thumb is that you can always give more but you cannot take away what has been given. Practitioners are best served by following a protocol to determine sensitivity and optimal dose for the particular desired effect by beginning at a lower dosage and titrating upward to the desired effect. To clarify, we have very small people who require very large doses and very large people who require very small doses. There is interest in developing predictive measures for ketamine’s depth of effect such as genotyping and clustering groups who respond differentially to ketamine. Easier is an empirical approach raising dosage in sessions appropriately to the actual response to ketamine. Clinical responses to ketamine based on personality rigidity and density of obsessionality are emerging as indicators loosely determining responsivity—loosely because there is no linearity to responses to ketamine for any diagnostic category. Rather, assessing individual sensitivity is the key to successful treatment (2).

These variables determine the nature and depth for the manifestation of ketamine’s effects as described as its Signature in Table 2.

**Table 2:** The ketamine signature—considered in relation to dosage and sensitivities of patients.

Rapid onset, rapid metabolism, dose-related escalation of effects, and degree of ego dissolution A time-out from usual identity and concerns enabling a reconstruction of being and letting go to resume life a bit differently, more connected and relaxed, a movement toward equanimity and balance. Opens to a vast array of experiences internally that are not predictable, sessions always varying, and generally disconnected or only vaguely related to ordinary mind—hence a period of liberation from our constrictions. An endorsement of our imaginative capacities—extraordinary experiences—and a relief from emotional distress that can lead to a recognition, that for example, my essence is not that of a depressed person. Out-of-body experience/Out of bounded time/Out of bounded space/Movement. An experience akin to meditation in its release and depth. Enables advances in the depth of meditative practices without ketamine and in our teaching of meditation. Reduction/elimination of external sensations—particularly visual, tactile and auditory/dose-related diminution of the sensory with expansion of the internal sensory, particularly visual. Synesthesia. Preservation of observer/monitor awareness or consciousness: The Guide—assisting us in the feeling of balance and imbalance. Ego dissolution/non-attachment/humility/cosmic consciousness/being small, less of us, which leads to the desire for being connected. Absence of negative emotions. A peaceful presence/self-acceptance without effort. No monsters in ketamine land. Trauma may appear in metaphorical formats and be released. Reduction of verbal thinking/heightening of the internal visual experience. Fluid connectivity and thematic movement engenders unique experiences. The mind roams freely while being observed and assessed levels of consciousness are enacted. Experience of being an Energy format. An Apprehension of Death from the perspective of Formlessness. Often a sense of having died and being at peace with death. The body dissolves and without form, there is only us as energy—a petit mort. Little ability to direct experience. Recall may be limited/dose dependent May be emotional with highly positive affect. Love as connectedness as well as self-appreciation. Sensual—some feel the experience to have sexuality internally. Can be disorienting, especially to the inexperienced, as at increasing dosage, there are full realties in which one is engaged. One may be entranced and happy there and never want to leave, never be able to leave, this is my life. Or long to escape, to return to my family (with a new appreciation of that connection) and fear that one will not return. Agitation may occur reflecting the enactment of the journey itself. Therapists do not have any idea whatsoever of the experience occurring and may make assumptions that lead to interventions based on the perception that their patient is suffering, only to hear on their return that it was the best experience of their life. Experience with KAP engenders in the therapist a rust in the process of the experience and its movement. This is always assisted by music for grounding and moving the ketamine experience on. Trust in this process is an essential part of the learning of doing this work. Indeed, there are difficult experiences, and these are grist for the ensuing therapeutic process, insight, awakened mind and body, and the working with trauma and its persistent effects. Nausea and less frequently vomiting are the major side effects. Hypertension is transient. Ketamine is a remarkably safe medicine. For many experienced psychedelic users, ketamine is the most powerful experience they have had. All the more reason to be careful in its use with those who are new to it. Ketamine, like all psychedelics, is transdiagnostic. Ketamine is remarkably effective for depression, suicidality, PTSD, OCD, menstrual syndromes, relationship issues, identity crises, and existential distress.

Amplifying the varieties of KAP work and thereby stimulating different aspects and the depth of subjective experience, we have developed and validated in clinical practice core protocols now for over 8 years. Utilizing our formulation of ketamine rapid-dissolving lozenges (RDTs), we determine appropriate dosages and titrate upward based on sensitivity and clinical considerations. Intramuscular injections (IM) are also administered in a dosage escalation format. Our practice includes supervised at-home sessions with our RDTs in our clinical program which supports the in-office therapy and is part of our efforts to make KAP affordable with quality of care. Other routes of administration are used selectively (2), as are different ketamine formats for different indications.

It is not the intention of this study to provide a manual for how to do KAP. That is a complex undertaking requiring training and supervision. It is our contention that the experience of the medicine is essential to understanding its effects on those treated. To date, we have had over 900 practitioners attend our 5-day experiential and didactic programs. Ongoing training, sharing, and collaboration are the essence of our development of KAP as an alternative clinical program. As the number of practitioners and our experiences with KAP grow, confidence in our hypothesis is strengthened. We have published a clinical review of 235 patients in 2019—See Dore et al. (2)—and are moving to a larger and more rigorous review of multiple KAP practices with many hundreds of KAP patients (see our KPA organization and KTC websites for more information on our programs). We have expanded KAP to work with adolescents and their families (82).

## Ketamine psychotherapy

Ketamine-assisted psychotherapy is an approach that allows for an integration of biological, psychological, and spiritual healing approaches (8). Established techniques and methodologies are being adapted to the Signature of the medicine and are in an unfolding, developmental process that applies to all psychedelic psychotherapies that are emerging into our alternative clinical practices. Psychodynamic, psychoanalytic, Internal Family Systems, EMDR, DBT, meditation, ritual, emotion-focused therapy, systems theory, strategic systems practices, Gestalt, Ericksonian hypnosis, a variety of approaches to trauma, social support, couples, and group psychotherapy—and more —are in use and in discussion among practitioners as to their employment and appropriateness for KAP. It is an exciting time to be a psychedelic psychotherapist.

With attention to the subjective nature of the ketamine process, KAP sessions tend to run about 3 h with further time for recovery before release from our office. Ketamine is ideally suited to a psychotherapy practice as its duration of significant effect tends to be 35-to 75 min, with peak experiences—including those of full ego dissolution—lasting about 20–40 min and then moving into lesser intensity followed by a period of drifting and then return to orientation. This range of time for effects enables us to begin with an hour or more of preliminary psychotherapy and with time for Integration and processing as the medicine wears off. Ketamine’s metabolism is remarkably consistent regardless of dose and tends to be replicated in duration per person. Various approaches can be taken to extend its timeframe for effect. Multiple sessions tend to be necessary and are designed per person and per diagnosis, severity, and psychodynamic presentation. Follow-up and near-term integrative sessions are essential for support of patients, their reframing of their stories, assistance with confusion that may emanate from such powerful experiences, and for the positive effects occurring from the relationship with the therapist—especially the arising of trust.

## The KAP process—facilitating the subjective experience

Upon absorption of ketamine, there is a release from the dominance of usual concerns, obsessions, and attitudes. Mind chatter reduces, the ego attenuates its hold, and there is a simultaneous release from the tension held in the body musculature. The Reichian concept of body armor and corresponding psychological defensive structures become relevant as individuals soften from these physical and mental protective structures, expanding into an experience of safety and emotional contact. An expansion of awareness that connects with emotions or memories held in deeper layers of consciousness ensues, depending on the depth of the experience. Lower doses allow for greater contact with the personal and more engagement by therapists throughout the experience. This is highly effective in group psychotherapy formats. Higher doses lead to full states of internally perceived realities and often experiences we characterize as transcendent and transpersonal (1).

In its effect on ordinary consciousness and its dosage-related attenuation, the non-grasping experience of ideal meditative states is achieved. The intensity of the experience of a state of formlessness and release from external sensation into the internal visual stream observed by a higher level of preserved consciousness augmented by the medicine tends to a full experience of oneself as an energy being. Movement of mind is a feature of ketamine journeying. Freed from form, the mind roams creating new realities and experiences that are generally unrelated to daily events, conflicts, and ways of being. This liberatory experience from the here-and-now fosters daily meditation practices. It leads to a fuller and broader experience of the self (54, 84–87).

The ketamine session provides therapeutic impact along many different axes. An experience of safety and defensive softening permits deeper contact with feeling states and aspects of the self that previously needed to be disconnected from for emotional survival. Reconnecting with these disavowed states and self-aspects in a field of safety and relational care allows for trauma metabolism and release that heals early wounds and integration through reconnection contributing to integrity and resilience. New insights and knowledge are frequently accessed both during and after sessions. Navigation of the ketamine experience cultivates the platform of an awareness state—a new or revived witnessing of one’s self—such as new views and attitudes, reframing, connection, affection and love, improvement in reality testing and sensitivity to the body and its sensations and states. The ‘letting go’ increases our capacity to notice and observe the symphony of qualia, the great show of being alive and sensate, interdependent, and connected, fostering mindfulness and loving kindness toward self, others and rooted in nature.

Another aspect of the inter-subjective space cultivated in the relational field is the complexity of a shared consciousness that contributes to new possibilities: The ability to be accurately seen, witnessed, felt, and responded to by a psychotherapist. The therapist listens and responds, in the present moment, and uses their full being as an instrument to listen and understand the unfolding process (88, 89). Such conditions, when achieved, provide the ideal conditions for trust, repair, emotional co-regulation, and psychological, spiritual, and social growth.

It is essential that the psychotherapy process is informed and guided by the subject’s own psyche. Reflective introjection—the capture and integration of new learning—its adoption operationally and as part of the self-concept—this seems the essence of growth and being. It is the opposite of compulsion by outside force. It is based on realization and persuasion. What has been come to be called ‘inner healing intelligence’ directs the unfolding of the experience. It reflects the poetry and knowledge held within. It also holds the need for justice, self-expression, the rectification of errors and untoward behavior, the sensation of freedom and liberation, and more. Foundational to this approach is a belief that all living systems possess an intrinsic capacity to heal, repair and blossom into full potential when adequate and appropriate conditions are provided—see Whitehead 1929 (90) as an early proponent of this organismic achievement of potential excellence. And Thompson representing the Santiago School of Varela as a later contributor (91).

Psychedelic-assisted psychotherapy thereby shifts the conventional psychotherapy approach and moves treatment toward a transdiagnostic healing paradigm aimed at building wellness, health, personal freedom, tolerance, and compassion for self and others—a sense of belonging, a movement toward the rebuilding or first acquisition of careful trust. In the psychedelic psychotherapy process corrective emotional experience co-mingles with the healing of wounds for a less pronounced impact on life. Living life as fully as possible, recognition of interdependency, the possibilities for our giving and receiving, our sharing and our collaboration in building a civilized culture, these are the values of an emancipating psychotherapeutic process. Ketamine-assisted psychotherapy in its best, ethical, and vital application moves us into this process of realizing who we are and how we wish to be.

## Illustrative case examples of the variety of the subjective in KAP practice

The following proper names are pseudonyms, and any details of identity are obscured for confidentiality. The patients provided their written informed consent to participate in their treatment and for anonymous inclusion of their experiences in this paper.

Assessment measures reported in these case examples are as follows: ACE—Adverse Childhood Experiences; Res—Resilience; BDI—Beck Depression Inventory; HAM-A-Hamilton Anxiety Scale; PCL-C—PTSD Checklist/Civilian; MEQ—Mystical Experiences Questionnaire; EDI—Ego Dissolution Inventory/Short.

## Case example: Gina—a psycholytic exemplar

*Keywords*: complex PTSD, major depression, low-dose ketamine, somatization

*Abstract*: A highly physically and emotionally life-long traumatized woman in her first SL session has a recollection of trauma and neglect that was physicalized. In the ketamine trance, a straight-forward suggestion by the therapist is received as an emotionally corrective experience. This is illustrative of ketamine’s potential to voluntarily loosen defensive protections against recollection, thereby allowing for relief from past trauma. We present this here as a portion of our work with this woman describing an aspect of ketamine’s rapid effect in opening healing doors.

We present the description of a first session for a 40-year-old horrendously traumatized woman. Diagnoses were PTSD (F43.10); Complex PTSD for which we use F43.9; Major Depression (F33.2), and Psychological Factors Affecting Physical Disorder (F54). She suffers from a rare and chronic, debilitating only recently diagnosed progressive systemic autoimmune disorder.

A second marriage to a physician resulted in two children and caring for his three children by his prior marriage. Gina is an RN in graduate school for family nurse practitioner (FNP), when her illness allows her to attend classes. The family situation is highly stressful with five children, an often absent husband, and a rural living location which constrains resources. Suicidal ideation without plan was frequent. Intake medications included duloxetine 60 mg; amitriptyline 25 mg and clonazepam 0.5 mg prn. A large variety of medications had been employed to treat her Blau syndrome with progressively diminishing respiration accompanied by GI and rheumatic symptoms. Maternal history includes cluster A personality disorder, sociopathy, and probable bipolar disorder. The father’s history suggests a significant personality disorder.

### Mental status exam

Gina is an above-average intelligent and vivacious woman, despite her obvious depression and fatigue from hypersomnolence. She suffers from pain and shortness of breath, as well as being overweight. Her presentation is always colorful and thoughtful. This optimism is what has carried her through the emotional and physical trauma of her life, which is now and has been extensive. Gina tends to take on too much and feel overwhelmed. There is a lack of adequate support, and her rural living situation leads to isolation and loneliness. She is a warm and friendly person. Symptoms of PTSD include involuntary recollections, distrust and inability to ask for help, periods of despair and hopelessness, social withdrawal, tearfulness, and resignation. She refers to herself as depressed, which includes an overlap of the PTSD symptoms. With all of this and the impact of her physical illness on her, she still does her best to be a loving, caring mother and is slowly making her way through a graduate program—somehow.

At intake, BDI was 44; HAM-A 48; PCL 70; ACE 9; and Resilience 7.

Treatment of Gina has been intermittently ongoing, punctuated by periods of disability and the now 16-month therapeutic contact has been benefitted by KAP sessions with in-office IM ketamine and at-home SL sessions—when possible. These provide moments of respite and relaxation—a time-out from stress that is constant otherwise.

This report is rather focused on an unusual emotionally corrective episode under ketamine’s influence at a low dose, classically called psycholytic treatment. It is presented as an informative example of the flexibility of the administration of ketamine when embedded in a psychotherapeutic practice.

After swallowing her first ever 100 mg lozenge and having held it for 15 min, she is feeling the effects of the ketamine. ‘That was awful. I could barely keep it in. It was like when my father stuffed mashed potatoes into my mouth choking me. I am always reminded of that. I was about seven and he left as a seaman, left me forever with my awful mother.’ I say as a matter of fact, ‘Your father is not here, he is dead. There are no mashed potatoes. It is just a lozenge’, she cries in relief. After the session, she describes to me all the ways in which that traumatic incident has marked her life: her fear of suffocation, of being given a bite of food by another’s offering, and her avoidance of situations in restaurants or around her table that trigger her memory as if it is happening and happening again and again. She repeats, ‘My father is dead, there are no mashed potatoes suffocating me. I am free.’

The trauma moves into the past. It is no longer alive in her. On my part, it comes out spontaneously, linked to her experience, stated blandly, without hesitation, as a deliberate cracking of the repetition of the horror. ‘It is not occurring now. You are safe. Your circumstances are entirely different. You have built a good life. Live it. Live in it. When the recollections come and result in your behavior, check it out, cut through the spell of the violence done to you.’ There is no need to say all of that. The ketamine has opened the door to accepting relief and letting go. All of that is contained in an instant of realization.

## Case example: Elena—a grief-induced obsessive-compulsive disorder

*Keywords*: OCD, bereavement, ketamine-assisted psychotherapy, tic disorder, PTSD, Germaphobia

*Abstract*: Elena is a now 59-year-old married woman who presented for ketamine-assisted psychotherapy citing depression, anxiety, insomnia, debilitating compulsions, and uncontrollable obsessions. She has been in KAP treatment now for some 15 months.

While her childhood was marked by parental abuse, neglect, and disrespect for her autonomy, and later on, sexual trauma, Elena was able to develop a capacity for warmth and independence. The consecutive illnesses and deaths of her two sisters and her deep involvement in their care created a desperate desire for control of minutiae and the projection of contagion with compulsive rituals and anxious, unstoppable rumination. She stated that she was living a routine without joy or purpose, compulsively serving others, not taking care of her own needs, and living in near constant grief.

Menopause and fearful self-imposed COVID restrictions were additional stressors. A familial tic disorder of variable penetrance would occur under stress and was more activated during low-dose ketamine sessions, often with spasms of the hands and feet, that would compromise her ability to experience ketamine’s effects as a relief from her suffering.

Nonetheless, with careful attention to her fear of loss of control and with her participation in decision-making on dosage, therapy has proceeded nearly linearly in the direction of a general lessening of control, reduction of OCD, processing of her trauma and losses, more pleasure in living, and appreciating herself. This has been affected by the intensity of the therapy, her trust in her therapist, and the acceptance of ketamine’s impact on letting go without harm occurring—this fostering an existential grasp of her inability to have prevented her sisters’ illnesses and deaths.

### Mental status exam on intake

Elena is of Mediterranean background and is presented as an anxious woman, wearing a mask and explicitly stating her fear of COVID. The previous day, she told us of a serious argument with her husband, who was feeling the stress of her insistence on his showering when he arrived home, her repetitive hand washing, obsessive cleaning and sanitizing, and their loss of intimacy. She spoke of feeling lonely in the marriage and of his irritability and distance since the recent death of his mother. Elena shared statements such as ‘My mind never stops running—a constant stream of noise, songs repeating on and on for days, repeated echoes of prior conversations, fears, worries, instructions, reminders repeatedly ‘. ‘I am clear on what I do not want to do, but not on what I want to do’. ‘I am just living a routine, no joy or purpose, just responsibilities, obligations, every day navigating the grief, constant stress and what feels like only moments of real rest’. ‘My mind feels like it is melting slowly. Simple tasks seem incredibly difficult—stress, worry, fear, memory issues.’

Exacerbation of her symptoms occurred during the course of her sister’s illness and became severe after her second sister died. The manifestations of her traumatic experience included some intrusive recollections of her sisters’ illnesses; a focus on that period that took up much of her mental space; bereavement; fear for herself and of illness, obsessional and compulsive concerns with hygiene and contagion with the impact of these concerns on others, especially her husband—and she was working at home due to COVID, anxiety, insomnia, and severe depression.

She denied current suicidal ideation, although she had thoughts without planning at earlier times in her life. Cognitive capacity was at an above-average level, and she was entirely responsive to interactions with a readiness to bond with the therapist. There was mild psychomotor agitation and significant insomnia with difficulty in falling asleep and maintaining sleep. She both spoke of fatigue and appeared fatigued. Elena was introspective and interested in knowing herself and seeking relief and treatment. Though avowedly anxious about beginning ketamine treatment, she was also hopeful and looked forward to its inception. There was no evidence of hallucinations, delusions, or organicity.

Elena was casually and appropriately dressed, with excessively good hygiene. She was struggling with menopausal symptoms, including frequent hot flashes. There were no medical illnesses save for the perplexing infrequent tic disorder usually manifested by repetitive heaving of her chest without respiratory distress. She spoke of a sense of her body always wanting to dance—an akathisia-like sensation. Substance use was limited for many years prior. Alcohol use was infrequent, and there was no history of tobacco use.

Medications were limited to trazadone, used episodically for sleep. Elena had been receiving acupuncture treatments.

### Diagnoses and assessments

On intake: PTSD F43.10, OCD F42, Major Depression F33.2, Psychophysiological Insomnia F51.04, Menopause, Unspecified tic disorder F95.5.

These Diagnoses have continued with remission of major depression and improvement in the degree of symptoms for PTSD, OCD, and insomnia. Menopause has been treated with HRT (hormone replacement therapy).

On Intake, BDI 34; HAM-A 30; PCL-C 56; ACE 4;Resilience 7.

Current: BDI 9; HAM-A 8; PCL-C 15

### Course

Elena’s fearfulness about loss of control, which is at the center of her PTSD and OCD complex, extended to the proposed ketamine session. With her participation in decision-making, we began carefully and at a low dose. She refused headphones wishing to remain in contact with us and fearing disorientation—to which we readily agreed. Her intention for this, her first experience of ketamine, was expressed as ‘I want to quiet my mind and smooth out’.

We began as is our usual procedure with a 100-mg rapid-dissolving tablet (RDT), the saliva containing the medicine, held for fifteen minutes, and swished. Her anxiety began to abate. At the twenty-minute evaluation of effect, she readily agreed to a second RDT, and the resulting saliva with its ketamine absorbed through the oral mucosa was held for another fifteen minutes. Elena spoke of feeling tingly all over her body and moved her arms and legs as if dancing. She commenced giggling and spoke of being in the cosmos. She said, ‘I feel really good, and I feel free. I feel powerful, grounded, solid and settled’. As the experience proceeded, she became calm and peaceful.

At the integration segment of the session, as the ketamine wore off, she expressed loving feelings toward her husband and processed her issues with handwashing and hygiene. She came to the understanding that these were manifestations of continuing her relationship and caring for her sisters, and in a certain way, they were a continuation of those relationships.

The second session commenced with enthusiasm and much less anxiety. In the two-week interval, her OCD symptoms had reduced—somewhat. We agreed to a higher dose of three RDTs, or 300 mg, as Elena requested going further into the trance. The result was feeling calm, her body rested and spacious. The movement disorder had been present for about twenty minutes during the peak of the ketamine’s concentration with spasmodic movements of her chest and legs. She spoke of it being ‘annoying when I try to relax’, and related other occurrences. We reviewed her family history, and I discussed her case with a movement disorder neurologist who considered it to be a familial movement disorder of Dyskinesia/Akathisia. Both her father and a sister were identified.

We prescribed RDTs for at-home sessions to be at a dose of 200 mg in the presence of her husband as security and provided our protocol for at-home sessions. This includes access to our playlists, music being essential for the ketamine experience as an anchor to this realm as a resource for disorientation, and providing the movement foundation for the ketamine experience to proceed without getting stuck in stasis (1).

As Elena’s ability to ‘let go’ took hold, so did her desire to leave her ordinary mind with its suffering and obsessions for an experience of freedom from this for the duration of a ketamine experience. She requested receiving an intramuscular (IM) injection of ketamine, and based on her response to the RDTs, we began with 70 mg. She related experiences of being on a ride like Disneyland’s Matterhorn; floating in water with a light; feeling ancient like an Egyptian in the red desert with the bright sun; and being in a movie with darkness all around. She never felt fear and enjoyed her visionary experiences. During her journey, she told a personification of her anxious part to let go. Her recitations of her journey were clear and detailed, as were her insights during the Integration portion of our session. ‘Loss and trauma bind people together’, she offered. ‘When I landed back in body, I relaxed. I traveled pretty far out. It was wonderful’.

Subsequent IM sessions have furthered her relief and reduced reactivity and OCD symptoms. She is no longer depressed and is resolved to take care of herself with her awareness of being trained to be duty-bound to others. She has had insight into her training in childhood to be afraid of disappointing her critical and punishing parents. Her fear of contagion has diminished with less pressure on her husband and a renewal of their closeness. They are planning a trip abroad for a month, which will also remove her from her managerial overwork due to duty and perfectionism, while hating her job, and being dependent on it financially. There have been periods of joy and a reduction in her COVID fears giving her more freedom to be out and about.

Elena has looked forward to her therapy experiences and her ketamine journeys. She has great gratitude and is much kinder to herself. Sessions are diminishing in frequency with over a month between them, as is the frequency of at-home sessions. In recent sessions, we have found a means to get past the spasms of hands and feet that have distracted her from her internal experience due to the discomfort they cause. This has occurred with an increase in the initial ketamine dosage to enable her to go deeper from the start, due primarily to her relaxation about loss of control. At the higher dose, the spasms are much reduced, and she has little experience of them. Elena has remarkable experiences of other realms and realities to which she has come to look forward, leaving her with a sense of leaving her past behind as an affliction rather than as memories that are integrated.

What remains as the goal of treatment is further reduction in OCD symptoms, and attention to self-care with reduction of her compulsion to work excessive hours. Elena is optimistic about herself and her future and is unequivocal in her view of the success of her KAP treatment.

## Case example: Anna—the effect of ketamine on developmental trauma and character defenses

*Keywords*: developmental trauma, dysthymia, low-dose ketamine, dissociation, attachment repair, inner healing intelligence

*Abstract*: Early attachment failures and developmental trauma that are characterized by neglect and emotional deprivation in early childhood are recalled and worked through during a low-dose ketamine-assisted psychotherapy. The patient’s inner healing intelligence and the psychedelic psychotherapy process is described.

Diagnosis: Persistent Depressive Disorder (F34.1).

Anna is a 33-year-old successful businesswoman struggling with chronic depression with bouts of anxiety and obsessive symptomatology. Patient had been involved in psychotherapy intermittently since college. She reported gaining insight but minimal relief of symptoms. At intake, medication included methylphenidate 20 mg daily, prescribed for ten years. She reported it had improved depressive symptoms, her focus, and attention.

There was a maternal history of a narcissistic personality disorder.

Her father’s history suggested the presence of an antisocial personality disorder.

Anna had recently started a relationship with a successful entrepreneur who referred her for treatment as he recognized the severity of her childhood and current emotional neglect. Anna felt that this current relationship was the most nurturing and successful relationship of her life. She was concerned for her ability to sustain it.

### Mental status exam

Anna presented as an attractive, petite woman, with a fragile, girl-like physique and a related manner. She was quiet and pensive with an intense gaze, quick, and intelligent in her thinking, in contrast with that other part of her that was expressed as a waif-like, almost ethereal, vanishing presence. Anna described bouts of depression, anhedonia, and difficulty concentrating. Periodically, she would become fearful and when stressed would become ruminative and obsessional. She reported difficulties in relationships and a prior pattern of tolerating abusive poor treatment by the men with whom she was involved.

There was no evidence of organicity, substance abuse, or alcoholism. There was no prior history of alternative medicine use.

### Course

Anna was seen in preparation for her first ketamine session. In the interest of beginning slowly and avoiding discomfort from disorientation, Anna was administered a single ketamine 100 mg RDT.

She proved sensitive to the medicine and experienced a lengthy journey, exceeding sixty minutes. Unusually for this dose of ketamine, Anna experienced visual phenomena and delighted in the colors and shapes she perceived. Expressing her pleasure, she comfortably navigated the experience with a sense of play and curiosity.

For her second session, Anna received 15 mg of ketamine intramuscularly. Within a few minutes, she entered a deep space in which she appeared young and fearful. Collapsing into a child-like, anxious state, she repeatedly asked for reassurance and giggled with pleasure and relief when I responded, feeling reassured I had not left her and indeed was near her and closely attending to her. In the trance state, she repeatedly felt a loss of her connection to me, promptly raising her fear of abandonment. This sequence of loss and reassurance occurred over the next twenty minutes or so. On integration, we focused on her historical narrative of loss and reconnection, with a recognition of how this had shaped her life.

One week later, we followed with an Integration session without ketamine. Anna declared she had an important breakthrough with an issue she had been struggling, which she attributed to her KAP work. She related that she had recently hired a couple to work in her business and could not fathom why there were numerous problems and complaints surrounding them. Immediately following the second ketamine session, she became clear that they were deceiving and manipulating her to take advantage. Confronting them, she found evidence of the couple’s mismanagement, promptly fired them, and took action to make amends with her clients.

On interpreting how she seemed to have been in denial of the couple’s neglect and incompetence, she wondered if this was a pattern of hers and of its origin. On reflection, Anna immediately saw it stemmed from a similar relationship dynamic with her mother. She described a neglectful, depriving woman who would rage, causing Anna to be confused about her responsibility, and feeling unloved and hurt. The randomness of the rage attacks and their damage left Anna uncertain of what she was entitled to emotionally and with a sense of naivete about others’ motives, with a persistent hope for being well-treated even when she was not. She went on to describe numerous examples where the cast of characters would change—previous bosses, boyfriends, and employees—but the plot was uncannily the same. A pattern of confusion around relationships with cons, abusers or crooks, situations where she would tolerate toxic treatment because she could not see clearly, thus rendering her ill equipped to leave the relationship and protect herself.

The defense of obscuring painful neglect and abuse as a means to sustain what she had deemed to be important relationships was brought to awareness and worked through during the ketamine session and the subsequent integrations. Significant attachment repair work occurred, and with it came a new ability to view others more clearly and advocate for herself. Surprisingly, Anna even appeared physically more substantial in her manner, physical presence, and presentation.

## Case example: Susan—extreme PTSD

*Keywords*: PTSD, involuntary recollections, traumatic depression, migraines

*Abstract*: A highly creative and successful woman in her mid-fifties is severely traumatized by the killing of her mother while holding her hand in a pedestrian motor vehicle accident. This is complicated by the betrayal and desertion of her wife in direct relationship to this horrific event. A chronic PTSD ensues with debilitating reliving of the event, periods of disability, and a terrible depression punctuated by episodes of suicidal ideation with possible intent. Intensive conservative care with multiple modalities and practitioners is unsuccessful, and the patient begins ketamine therapy. Over a few years of time, there is a progression of alleviation of the severity of symptoms and leads to a partial resumption of life activities. We present her extraordinary exemplary ketamine experiences in which there are themes of healing and relief.

*Background*: S is a now 62-year-old gay woman and successful attorney who suffered an unimaginable horror. She left the hospital where her father was recovering from a myocardial infarction, was holding her mother’s hand, and was crossing the hospital parking lot when a car veered out of the lot. It struck her mother, nearly decapitating her and dragging her under the cover for a long distance before stopping. The driver was unaware. S′ mother died some hours later in the hospital ER. S was mildly injured.

S was married at the time, and her wife took the opportunity to confess a long-standing affair with another woman, helped S to be hospitalized, and left her for the other woman. While S was there, she stripped their house and instituted divorce proceedings. S′ siblings were traumatized but lived at a distance, had not witnessed the event directly, and offered some support. S had a history years prior of a pituitary tumor with a botched surgery that required repair of her sinuses as she was leaking spinal fluid and was left with terrible, near-daily migraines.

### Mental status exam

Prior to the butchery of her mother, S had been a vibrant, social, and joyful woman. When I met her about 3 years after the atrocity, she was ravaged by intense symptoms of PTSD, with frequent involuntary repetitions of the event, awful depression, anhedonia, and a collapse of any life meaning. Despite this and her migraines, she had managed to return to her legal duties. To cap it off, the insurance company had engaged in legal actions to block recovery of her entitlement, which led to legal proceedings that would increase the PTSD symptoms and disable her. This, anniversaries, crossing the street, and any sudden events would all jar her into reenactments and despair. The reliving of the events was vivid and prolonged, with exhaustion, confusion, and retreat being consequences. She admitted to suicidal thoughts and some degree of planning, these occurring especially when symptoms were obliterative of her own life force. S. had become solitary and in retreat, giving up her highly creative life, her friendships, and her athleticism. She struggled to attend work and had only limited hours for functionality, pressing herself beyond her stamina. Her anger was present but subdued. There were guilty thoughts, mostly focused on leading her mother into the crosswalk. The trauma of her wife’s rejection and betrayal was profound, contributing to her loss of interest in relationship and the absence of any libido or desire for life activities. When we began with our ketamine work, S was seeing a psychiatrist, a therapist, and an EMDR specialist. She was on multiple medications, including benzos and antidepressants, which were ineffective, as had been others prior to the then-current regimen.

*Diagnoses*: PTSD-severe (F43.10); Major Depression (F33.1); suicidal ideation and frequent migraines,

BDI was 44; MADRS 33; PCL 70,HAM-A 30; ACE 3; Resilience 10.

Her measures now some six years later reflect her recovery: BDI 13; Ham-A 15; PCL 34, MEQ 21; EDI 40. The latter two measures reflect the positive impact of mystical/ego-dissolving experiences with ketamine.

Medications included escitalopram, methylphenidate, temazepam, oxycodone 10 mg—frequency depending on near daily migraines, and medical marijuana for sleep prn. There was no history of substance abuse or use, nor was there any consumption of alcohol. During the course of the six years of our treatment, she used olanzapine 2.5 mg for periods of time when unusually overwhelmed by involuntary recollection and suicidal ideation; she was prescribed bupropion and midrin for migraines.

### Course

We began with lozenge sessions, established her sensitivity, and continued with regularly spaced IM sessions and at-home ketamine lozenge sessions. These produced respites and, over time, a diminution in the intrusive recollections. The depression and anhedonia would improve a bit, and then an event would occur that would set her back. Of note was that ketamine reduced the frequency of her migraines—this is not always the case.

The IM sessions would invariably contain major elements of her trauma, yet with a spirit of overcoming the horror and a celebration of her and her mother’s closeness. They were always thematic, beautiful, and affirming of S’s spirit and the possibility of living with the great grief and horror—and eventually moving into a more vibrant life—a reclamation while the stream of grief and shock would always continue, with the stream of living growing slowly in intensity. That has happened, but not fully, and we are still engaged in the recovery and the resumption of some level of trust in humans and life itself in each moment. Our relationship is part of that reclamation, which I am pleased to report. I am her therapist, but in the remarkable nature of our collaboration and the depth of our mutual knowledge of loss—I lost my eldest son at sixteen to leukemia—we are together and in understanding.

One of her extraordinary journeys:

*I was kneeling over my mom and her mangled, twisted body. The wreckage of her. But underneath the rubble and blood and mangled limbs was her heart. Perfectly intact. Fertile ground for*. A*nd a baby tree began to grow over that heart. Its roots surrounded it as if making a protective cage. The canopy of the tree was billowing like a verdant cloud. Larger and larger. The limbs stretching toward the sky gave way to the branches at its tips. Those branches gave way to hands, which unfurled to release dragonflies and red hearts that showered down upon my mother. The dragonflies were dive-bombing a one-dimensional army below of some sort of midget like figures. If these figures looked up and caught one of the hearts, they were transformed into a three-dimensional being. I felt alive and happy. We were as one.*

S. has made much of a recovery, slowly and with exacerbations, especially brought on by circumstantial events that were triggers to the trauma. This was especially the case with the protracted insurance company’s withholding and litigiousness, which drew in all her practitioners. She has partially resumed her remarkable creative life as a photographer and musician, but not socially at the level prior to her trauma, which was multifaceted and included betrayal and a profound rupture of trust during the worst moment of her life.

## Disturbances in the field—commercialization and its cost to care and the expression of the subjective

All human expression and interaction occur within a context of values—internal and external. Values are what shape religion, relationships, societies, and certainly that small subset of human endeavor we call psychotherapy (92).

To be clear, in relationship to evolving social consciousness, there has been a near steady state of expunging prejudice from psychotherapy since its origin. Taking a view of that history, it is rife in its relationship to misogyny and male dominance, racism, repression, forced incarceration, gender and ethnic prejudice, and to Pharma and academic influence and control. This applies not just to the legally empowered psychiatrist but to all the allied professions. It is one of relativism to times and culture. Unfortunately, historically, our profession has not served as an absolute pinnacle of wisdom and loving-kindness. There is still much to be realized with ongoing controversy about the social underpinnings of trauma and prejudice on mental health. Untying this thorny knot leads to the weaving of the quilt of civilization. A great effort and commitment to deep inquiry into the awareness of prejudice are necessary for the transformation of consciousness. That inquiry is into our being as a species, as planetary inhabitants, as participants and creators of social formations, as to the impact of trauma, illness, and loss, as to privilege and the acquisition of privilege, as to greed and altruism, as to love and hate. That inquiry is into the heart of our subjectivity—into the heart of how we choose to live and work.

Ketamine and other psychedelic medicines potentially offer an experiential challenge to cultural dominance: the determination of what is deemed appropriate—the prejudice and its enforcement against full personal and social exploration that serves social control, consciously and implicitly (11, 93). The invitation is for clarity, pleasure, and wellness—plus imagination, exploration of the rules of the social game, and the desire to change rules that suppress freedom of mind and behavior (70, 94). It is a claim made here that set and setting are crucial to the progressive nature of psychedelic medicine use. For there can be misuse and abuse, which certainly occurs and has occurred (95). Ethical practice is essential to building trust and relationship. Indeed, there have been several surveys that present a view of personal changes made that are impactful on cultural views and behavior, as they are uplifting, ego-dissolving, and relieving of prejudices (61, 96).

We again return to values. The practice of psychedelic and ketamine medicine is not sacrosanct. Our primal addiction to money is too often in evidence. That addiction is both personalized and institutionalized. The War on Drugs drove psychedelic medicine out of its relatively inexpensive manufacture and limited distribution into monetization and an underground economy, which in fact increased the then-illegalized consumption of these medicines. The use of alternative medicines that had proven so effective and revolutionary to the practice of psychotherapy/psychiatry was eliminated or went underground (5–7). Moving out of Schedule I—and hopefully soon into prescriptive use—has proven expansive and arduous. Public awareness of the benefits of psychedelics in part has related to the legalization of marijuana in so many states. Add to that the interest generated by great results clinically (for Ketamine; MAPS and MDMA; Psilocybin), popular writing, and massive underground use that belies the propaganda of harm that has been reducing in scope and frequency. There is a sense that psychedelic medicines are in—and without accurate figures [few people confess to the National Institute on Drug Abuse (NIDA)], it is our distinct impression that public use has increased massively and is worldwide. For ketamine, NIDA estimated 0.13% of the US population had an experience in 2019 (97). Less formal sites estimated 3 million people aged 12 or older had used ketamine in the US as of 2015. For reference, MDMA use was put at 0.9% of the population (97). Young adults predominated for both.

Popular consumption brings with it great concerns. Educated use, a reverence for these powerful medicines, careful attention to set and setting, a sense of a broad and unofficial community that watches out for misuse and harm—all of this needs development. There are multiple causes for psychedelic casualties, from polypharmacy to ketamine. ER visits are most often associated with multiple substance use, newly introduced and untested substances, adulterated substances purchased in unreliable illicity, dependency, social and relationship consequences, and self-medication. There tends to be a lack of attention to the underlying suffering, confusion, disorientation, and obsessive use that may engender negative alterations of consciousness and hazards to emotional and mental health. For a full view of this, see Erowid.org.

Ketamine has an unfortunate reputation as a street drug—Special K, K holing, K, Vitamin K, etc. It is both necessary and ethical to provide ketamine clinically within a rigorous standard of care. Ketamine dependency occurs with cognitive, emotional, and physical consequences (98). And there are new related substances—analogs—that have come into use and more will come—that have significant dependency problems and arise with little if any information on safety (42). As above, it appears that ketamine has no more street use than any other alternative medicine, but its negative reputation tends to undermine public perception. That is changing, especially as ketamine’s value is established by its positive impact on mental health.

Thus far, in clinical practice with KAP, we have not witnessed diversion or drug seeking during our treatment. Close attention to prescriptions and amounts prescribed, observing use patterns, watching for overuse, utilizing non-abusable methods such as lozenges, and restricting IM use in the office—these are all means to prevent misuse of ketamine coming out of the clinical setting.

Ketamine is a very desirable medicine, but there are limited KAP resources for depressed and traumatized people. Issues of cost, access to care, outreach with telepsychiatry to those not served, all are important considerations for expanding KAP’s availability and clinical use. There is still no insurance coverage specific to ketamine. Therefore, financial strategies that promise ketamine’s use at a lower cost are proliferating. Unfortunately, this tends to be a misrepresentation. Adding profits to the cost of care reduces the amount of care delivered, promotes promiscuous prescribing, lessens the amount of time devoted to care by practitioners, impairs the quality and training of practitioners, diminishes the scrutiny as to who is being prescribed ketamine, and reduces attention to their overall mental health needs. All of this tends to attenuate the overall quality of care and increase the risk to patients. This certainly emphasizes the need for strategies, such as insurance availability for ketamine therapy, that increase the availability and affordability of this most desirable medicine.

## Conclusion

We have presented KAP as establishing an intensive and complex process for treating the whole person. Ketamine’s alteration of consciousness as a necessary and component ‘effect’ for a healing process to occur has been delineated. Understanding ketamine’s spectrum of psychoactive to psychedelic manifestations as ‘effects’ would unite the field of ketamine work. As a ‘side effect’, the denial of the subjective is implicit. A ‘side effect’ tends to be a label attributed usually to an undesirable aspect of a medicine. In the case of ketamine, it serves to retain the use of ketamine as a drug, eliminating inquiry into the nature of the recipient’s experience and the possibility of a psychotherapeutic process integrating that experience into mind and life changes. ‘Effect’ throws into bold relief the deep potentiality of ketamine as an agent promoting individual and relational processing—the impregnable value of the subjective.

The obvious limitation to our hypothesis moving to ‘proven’ is the early phase of KAP work and the requirement for more clinical studies to amass data as to outcomes. The varying nature of KAP therapies and practitioners may well impact this. So too will dosage and administration variations, which no doubt will continue to make correlations and comparisons difficult. Standardization of data collection as per the Ketamine Psychotherapy Association’s Redcap format will help if widely adopted.

A major problem confronting psychedelic-assisted psychotherapy, KAP, and arising clinic consortiums is the dearth of trained practitioners. Building clinic clusters in a rush without the presence of trained personnel diminishes the quality of care and the ability to conduct true psychiatric/psychotherapy practice. Ketamine is not for everybody, not for all maladies, and needs to be embedded in a tailored approach for the patient, who presents with their own specific complexity and matrix. It is not cookie-cutter work. People suffering in their myriad ways who arrive at our doors require full evaluation and the provision of personalized care with all the necessary psychiatric and psychotherapy resources. Ketamine is not a stand-alone medicine and needs to be part of a full-spectrum approach.

KAP skills take time to nurture and develop in each of us and in the clinics we build. The same is and will be true for MDMA and psilocybin practices as they arise. And the demand for alternative medicines as psychotherapies will only grow as their effectiveness and renown become known. It is important for practitioners to earn a decent living, and while doing so, it is an imperative to keep costs low, expand access to care, and support each other in the learning process of these potent and remarkable therapeutics.

Unfortunately, the cost of accessing new medicines as they are approved appears to be unreachable for the bulk of the population. Insurance reimbursement hopefully will arise, but insurance has been notoriously poor in reimbursement for psychiatric and psychotherapy. Incorporation into national health services would be remedies where these exist and are of quality. High prices for desirable medicines move people toward the unlicensed underground or to personal, unsupervised use.

From the KAP perspective, it is our commitment to set the standard for quality of care. To this aim, we are entirely devoted (99). Increasingly, the recognition of the potency of KAP provided with a high standard of care moves us to prove our hypothesis to the community at large. For those of us practicing KAP, we are already aware of the remarkable nature of these experiences for both patients and practitioners, and we are its advocates. This is the hypothesis we are engaged in proving to be true.

## Data availability statement

The original contributions presented in the study are included in the article/supplementary material, further inquiries can be directed to the corresponding author.

## Ethics statement

Written informed consent was obtained from the individual(s) for the publication of any potentially identifiable images or data included in this article. Written informed consent was obtained from each participant for the publication of these case reports.

## Author contributions

All authors listed have made a substantial, direct, and intellectual contribution to the work and approved it for publication.
